# Priming of human monocytes by β-glucan and obesity-associated factors: modifications to global DNA methylation, gene expression, phenotype and function

**DOI:** 10.3389/fimmu.2026.1825431

**Published:** 2026-07-20

**Authors:** Ben J. Topham, Barry D. Hock, George A. R. Wiggins, Louisa V. Ashby, Nicholas J. Magon, Elisabeth Phillips, Emma K. C. Symonds, Kirsty M. Danielson, Margaret J. Currie

**Affiliations:** 1 Mackenzie Cancer Research Group, Department of Pathology & Molecular Medicine, University of Otago Christchurch, Christchurch, New Zealand; 2 Haematology Research Group, Department of Pathology & Molecular Medicine, University of Otago Christchurch, Christchurch, New Zealand; 3 Mātai Hāora - Centre for Redox Biology and Medicine, Department of Pathology & Molecular Medicine, University of Otago Christchurch, Christchurch, New Zealand; 4 Department of Surgery and Critical Care, University of Otago Wellington, Wellington, New Zealand

**Keywords:** DNA methylation, gene expression, immune function, innate priming, obesity-related compounds

## Abstract

**Introduction:**

Monocytes play a critical role in regulating immune response to exogenous and endogenous challenge. Recent studies have shown exogenous stimuli can induce metabolic, epigenetic and phenotypic changes in monocytes that reprogramme or ‘prime’ them to react with an altered response to subsequent stimuli. Obesity is associated with immune dysfunction. However, the ability of endogenous compounds associated with obesity to prime human monocytes and thereby modulate subsequent response remains to be investigated.

**Methods and results:**

In this study, human monocytes from non-obese males were utilized. Monocytes exposed to primary exogenous stimuli (β-glucan), but not endogenous stimuli (oxLDL, leptin, 17β-estradiol), demonstrated an altered phenotype (increased CD11b, PD-L1 expression) in response to SK-MEL-28 melanoma cells. In contrast, monocytes primed with oxLDL, but not β-glucan, exhibited an increased rate of superoxide production in response to secondary stimulation with phorbol 12-myristate 13-acetate, and enhanced matrix metalloprotease 9 activity in response to secondary stimulation with lipopolysaccharide. Bulk RNAseq analysis of non-primed monocytes and monocytes primed with 17β-estradiol or oxLDL also showed sustained differential expression of genes related to immune function, metabolism and protein modification. Moreover, mass spectrometry analysis showed that monocytes primed with β-glucan or oxLDL exhibited sustained modifications to global DNA methylation.

**Conclusions:**

These results provide the first steps in understanding the functional consequences of monocyte reprogramming by obesity-related factors, and the potential pathways that govern them.

## Introduction

Monocytes play a central role in the regulation of the immune system. Depending on environmental cues, monocytes can either inhibit or promote immune responses (1–5). Recent research has shown that monocytes can acquire a *de facto* ‘memory’ of a primary stimulant through metabolic and epigenetic reprogramming, which results in long lasting changes in phenotype and altered response(s) to subsequent stimuli - a characteristic known as ‘innate priming’ (6–11).

Exogenous compounds have the capacity to heighten monocyte function against secondary stimuli (6–9). A recent study using a murine model provided direct evidence that innate priming of macrophages with whole β-glucan particles (WGP) can improve control of metastatic burden (9). Importantly, priming of macrophages was achieved without inducing systemic inflammation, minimizing exacerbation of inflammatory disorders (9, 12, 13).

Obesity is associated with immune dysfunction and the increased incidence and advancement of cancer, infection and autoimmunity (14–16). The occurrence of obesity is associated with significant changes in metabolism and the associated biosynthesis and release of potentially immunomodulatory compounds (16–18). However, it remains unclear how altered metabolic and hormonal profiles associated with obesity may affect the immune system at the level of individual cell types such as monocytes and thereby influence immune response to disease.

Endogenous compounds can also induce innate priming, with monocytes primed by oxidized low-density lipoprotein (oxLDL) showing increased production of reactive oxygen species (ROS) and cytokines, increased glycolytic metabolic activity and enhanced presentation of scavenger receptors (7, 19). In addition to oxLDL, aldosterone, uric acid and butyrate have been demonstrated to prime macrophages (7, 20–22). However, the extent on how endogenous priming alters monocyte function, particularly in different disease settings such as cancer, remains to be fully examined. These studies raise the question of whether circulating metabolites and hormones that are elevated during obesity could stimulate endogenous priming of monocytes, heightening response to secondary challenge.

In this *in vitro* study, we used human monocytes from non-obese males to compare the priming effects of a well-characterized exogenous compound (β-glucan) with those of endogenous compounds associated with obesity (oxLDL, leptin, 17β-estradiol) (23–25). We show that priming of human monocytes with β-glucan increased expression of CD11b and PD-L1 in response to subsequent stimulation by indirect contact with SK-MEL-28 melanoma cells. Comparatively, priming with an endogenous compound (oxLDL) altered monocyte function by enhancing superoxide production and MMP9 activity after secondary stimulation with phorbol 12-myristate 13-acetate (PMA) and lipopolysaccharide (LPS), respectively. Moreover, human monocytes primed with oxLDL or 17β-estradiol showed differential expression of genes related to metabolic and epigenetic reprogramming, and these gene expression changes persisted for at least six days after initial priming. Additionally, oxLDL primed monocytes had increased 5-methylcytidine levels, while β-glucan primed monocytes had decreased had 5-hydroxymethylcytidine levels, providing direct evidence that priming is associated with global DNA methylation levels in human monocytes.

## Materials and methods

Ethical approval for this study was obtained from the University of Otago Human Ethics Committee (H23/021).

### Study participants

Volunteers were eligible for the study if they were over 18 years of age, able to provide informed consent, had not previously received treatment for cancer, were not anemic (blood hemoglobin count >90 g/L), had not been vaccinated and were symptom free from illness for a minimum of three weeks prior to blood donation. Volunteers were only approached if they were male sex and not classified as obese. Volunteers were classified as obese and excluded from the study if they were >30 kg/m^2^ (BMI), >0.9 (waist: hip ratio) or >25% fat mass (impedance analysis) (26).

### Blood collection and isolation of PBMCs

Peripheral blood was drawn into 10 mL sodium heparin vacutainer tubes (Becton Dickinson, Auckland, New Zealand) by phlebotomists. PBMCs were harvested from the blood using SepMate PBMC Isolation tubes (StemCell Technologies, Tullamarine, Australia) and lymphoprep density gradient centrifugation and counted. Monocytes were then isolated from PBMCs through seeding cells at 4x10^6^/mL in RPMI 1640 + 2mM GlutaMax (Thermofisher Scientific, New York USA) + 1% Penicillin-Streptomycin (P/S) (Thermofisher Scientific, New York USA) media in a Nunclon Delta-treated plate (Thermofisher Scientific, Suzhou, China) and incubating for 2 hours at 37 °C, 5% CO_2_. Monocyte purity following culture was confirmed using CD64 expression (**Supplementary Material 1**).

### Priming of monocytes

**Figure 1:**
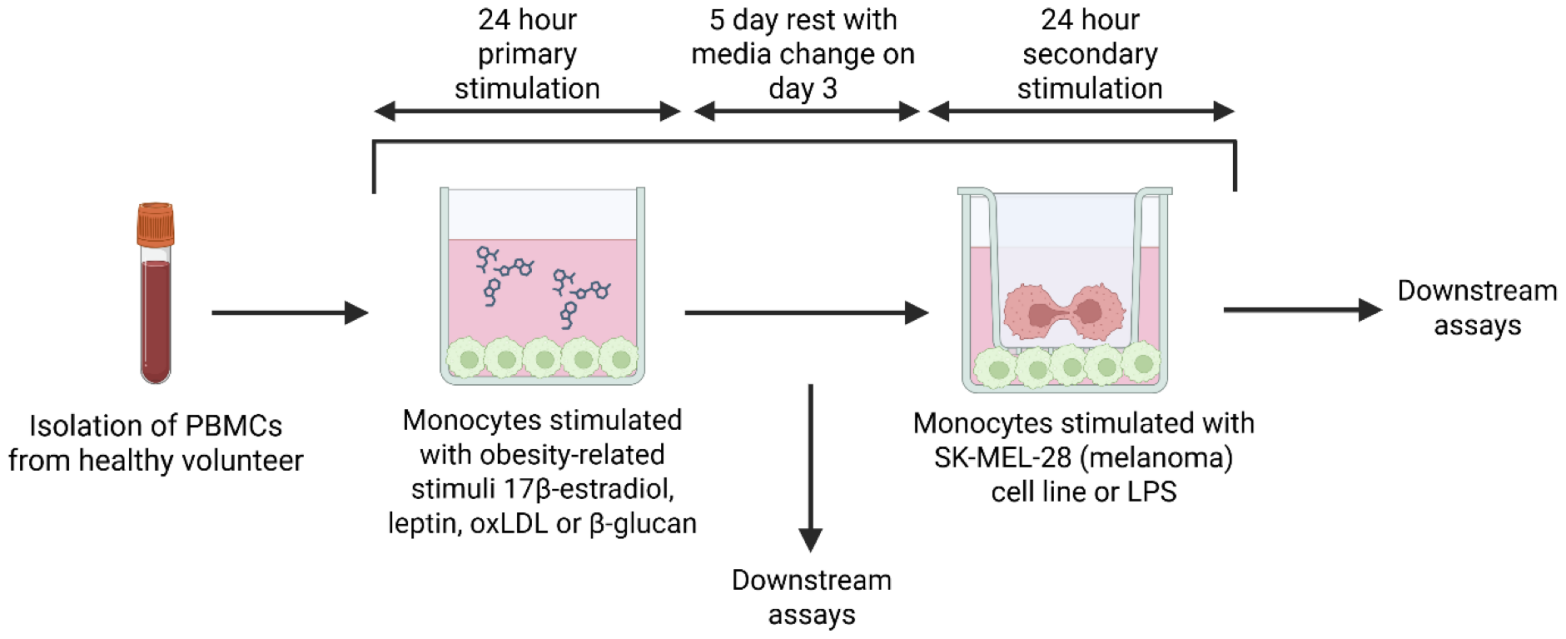
Model for priming monocytes with different primary stimuli followed by rechallenge with distinct secondary stimulants. Monocytes were isolated from PBMCs and primed for 24 hours with either media alone, or media with primary stimulants 5 µg/mL β-glucan, 330 pg/mL 17β-estradiol, 100 ng/mL leptin or 10 µg/mL oxLDL. Monocytes were then rested for five days in fresh media, with a media change on day three. On day six, monocytes were rechallenged with secondary stimulants 25 ng/mL LPS or indirect contact with SK-MEL-28 melanoma cell line (9.02x10^3^ cells) for 24 hours. Supernatant and/or cell samples were taken pre- and post-secondary challenge for downstream analysis. Created in BioRender. Topham, B. (2026) https://BioRender.com/q9y0krq.

Monocytes were cultured with RPMI 1640 + 2mM GlutaMax + 1% P/S + 10% hormone-stripped heat-inactivated FBS medium (Gibco, Missouri USA) or media supplemented with (i) 5 µg/mL β-1,3 Glucan (Sigma-Aldrich, Missouri USA), (ii) 330 pg/mL 17β-estradiol (Sigma-Aldrich, Missouri USA), (iii) 100 ng/mL leptin (Sigma-Aldrich, Missouri USA) or (iv) 10 µg/mL oxLDL (Invitrogen, Oregon USA). β-glucan is frequently used to study monocyte priming (7, 19, 27), while the remaining compounds are upregulated during obesity. Stimulant concentrations are within achievable physiological concentrations and/or previously used at similar concentrations within other immune studies (7, 23–25, 28–34). FBS was stripped of steroid hormones using activated-charcoal (35). Monocytes were then incubated for 24 hours at 37 °C 5% CO_2,_ before media supplemented with priming compounds was replaced with fresh media. Monocytes were then allowed to rest for five days, with fresh media replacement on day three. On day six monocytes were rechallenged with either media alone or media supplemented with (i) 25 ng/mL LPS (Sigma-Aldrich, Missouri USA) or (ii) SK-MEL-28 melanoma cancer cells in 0.4 µm transwells inserts (9.02x10^3^ cells) (cellQART, Northeim, Germany) for 24 hours. Schematic of priming model shown in **Figure 1**.

### Immunocytochemistry

Monocytes were fixed with methanol and stained with DAPI (Invitrogen, Oregon USA). Cellular nuclei were imaged with ImageXpress^®^ PICO (Molecular Devices, California USA) using the MD Cell ReporterXpress software (Molecular Devices, California USA) and count was performed using the ReporterXpress software.

### Flow cytometry

Primary and secondary stimulated monocytes were uplifted on day seven with Accutase (Sigma-Aldrich, Missouri USA) and concentrated to 1 x 10^6^ cells/mL. Cells were incubated with Zombie NIR (BioLegend, California, USA) and 2.5% human Fc blocked (BioLegend, California, USA). Monocytes were then stained with fluorescent antibodies TLR: AF700, CD11b:BB515, CD206:PE, PD-L1:PE-CF594, CD86:BB700, HLA-DR: BV480, CD163:BV421, CD36:APC and CD64:BV605 (CD64 and CD14; BioLegend, California, USA. TL4; Invitrogen, New York USA. Remaining antibodies; BD Biosciences, Tatabanya, Hungary). After staining, cells were fixed in 0.67% PBS Paraformaldehyde (pH 7.4) (Thermofisher Scientific, Massachusetts USA). Fluorescence was measured using 3-laser (violet-blue-red) Cytek Aurora flow cytometer (Cytek Biosciences Inc., California USA). Data unmixing was performed in SpectroFlo (version 2.0, Cytek Biosciences Inc., California USA) to identify fluorescence spectral signatures for each fluorophore, correcting for spill over. After compensation, droplets were removed and live cells were gated (**Supplementary Material 1**), before the geometric mean fluorescence intensity (MFI) was calculated using FlowJo software (version 10.8, BD Life Sciences, Oregon, USA).

### Superoxide production

Monocytes were seeded and primed within 96-well plates. On day six, media was replaced with RPMI 1640 without phenol red (Thermofisher Scientific, Paisley, Scotland) + 20 µg/mL catalase (derived from bovine liver, Sigma-Aldrich, Missouri USA) + 40 µM cytochrome C (derived from equine heart, Sigma-Aldrich, Missouri USA). Plates were placed into a Biotek Synergy Neo2 plate reader (Agilent, Vermont USA) warmed to 37 °C, and absorbance (A) of reduced cytochrome C was measured at 550nm, at 2-minute intervals for an hour after addition of 50 ng/mL PMA (Sigma-Aldrich, Missouri USA). Cells in each well were fixed with methanol (Sigma-Aldrich, Missouri USA) and counted, so that rates could be normalized to cell number. Maximum rate of change was determined using the steepest portion of the absorbance curve, that was continuous for a minimal period of 15 minutes. Cytochrome C reduction is directly stoichiometric with superoxide concentration (36), and the absorbance change recorded in the plate was calibrated against a 1mL cuvette. Beers law was applied using Ɛ_550_21,100 M^-1^cm^-1^ for cytochrome C reduction (37). The addition of 20 µg/mL superoxide dismutase (derived from bovine erythrocytes, Sigma-Aldrich, Missouri USA) to wells containing monocytes, catalase, cytochrome C and PMA, suppressed the increase of absorbance confirming the specificity for superoxide.

### MMP9 activity

Monocytes were primed and cultured for five days, before the media were replaced with virus production-serum free medium (VP-SFM) (Gibco, Paisley, Scotland) + 20mL/L Glutamine (Gibco, Paisley, Scotland) + 1% P/S alone or VP-SFM supplemented with 25 ng/mL LPS. VP-SFM is a low protein medium that does not require the presence of FBS to support cell growth. After 24 hours of secondary stimulation, the conditioned media was collected. Conditioned media was diluted 1:4 with non-reducing sample buffer and MMP9 activity was measured using zymography with 20% gelatin (Fisher Scientific, New Jersey USA) containing resolving gel and a 3% bis acrylamide (Sigma-Aldrich, Missouri USA) stacking gel. MMP9 was activated with 50 mM Tris-HCl (pH 7.4) (Roche, Mannheim, Germany) + 9.9 mM CaCl_2_ (Sigma-Aldrich, Missouri, USA) and incubated overnight with gentle mixing at 37 °C. Gels were stained with Coomassie brilliant blue (Sigma-Aldrich, Darmstadt Germany) and imaged using Q9 Alliance Imager (Uvitec, Cambridge England). Bands were analyzed using Nine Alliance Q9 software (Uvitec, Cambridge England). Background subtraction was performed using the rolling ball approach and band volumes were normalized by cell count.

### RNA sequencing

RNA was extracted from primed monocytes on day six using RNeasy Mini Kit (Qiagen, Maryland USA). RNA was treated with DNase I (New England Biolabs, Massachusetts USA) and samples from 10x independent donors for each condition with no technical repeats (40 samples total) were sent to Lincoln Genomics (Lincoln, New Zealand) for bulk RNA sequencing. Samples were depleted of rRNA and then processed with NEBNext Ultra II Directional RNA Library Prep (New England Biolabs, Massachusetts USA). The libraries were pair-end sequenced at 100bp using the MGI G400 sequencer (MGI Tech, Riga Latvia), producing >800 million reads, which is equivalent to approximately 40 million pair-end 100bp reads per library.

Sequencing quality for each sample was analyzed using FastQC (version 12.1 (38)). Adapters and barcodes were removed from sequences using Trimmomatic (version 0.39 (39)). Fastq files were aligned to the human genome (GRCh38-hg38) using the STAR (version 2.7.3a) aligner (40). The reference genome was first index using the Gencode GRCh38 annotation (version 44 (41). If a gene did not achieve a minimum of 10 counts in any sample, it was removed. A summary of quality control is reported in **Supplementary Material 2**. Differential expression of genes across samples was analyzed through applying a generalized linear model using DESeq2 (version 1.33.0 (42). Sample matrix detailing condition and batch (biological donor) was incorporated into the DESeq2 pipeline and batch effect (**Supplementary Material 2**) was corrected for. Comparisons were made between media and priming condition (17β-estradiol, leptin or oxLDL) pooled (across donors) differential expression using Wald test with Benjamin-Hochberg correction for multiple comparisons (42), and graphed as volcano plots. Log2 fold change cut off >0.5 or < -0.5 and adjusted p value threshold of ≤ 0.05 was applied for identification of significant differentially expressed genes. Differentially expressed genes (DEG) that reached the fold change cut off is shown in **Supplementary Table 1**, while full DEG is shown in **Supplementary Table 2**.

### Mass spectrometry

DNA was extracted from primed monocytes on day six using DNeasy DNA and tissue kit (Qiagen, Maryland USA). Nucleotides were liberated using a nucleotide digestion kit (New England Biolabs, Massachusetts USA), and samples were spiked using heavy isotopes of 85 fmoles of cytidine (^13^C, ^15^N_2_), 5 fmoles of 5-methylcytidine (^13^C, ^15^N_2_) and 0.13 fmoles of 5-hydroxymethylcytidine (^13^C, ^15^N_2_). Cytidine species were measured using a QTRAP^®^6500 mass spectrometer (SCIEX, Massachusetts USA) and were interpolated from standards through relating light-to-heavy isotopes peak area ratio to standard curves. Species were then presented as the percentage of total cytidine species (43).

### Statistical analysis

All statistical analysis was performed on GraphPad Prism (version 10.4.1, GraphPad, California USA), unless stated otherwise. All experiments were completed in biological triplicates at a minimum, and analysis was performed using mean ± standard error of the mean (SEM). Normality of data distribution was tested with Shapiro-Wilk normality test.

If data passed normality, multiple comparisons were tested using RM one-way ANOVA and Dunnett’s multiple comparison test with single pooled variance. If data did not pass normality, multiple comparisons were tested with Friedman tests and Dunn’s multiple comparisons test.

## Results

### Priming increases monocyte surface marker expression in response to subsequent stimulation by indirect contact with SK-MEL-28 melanoma cells.

**Figure 2:**
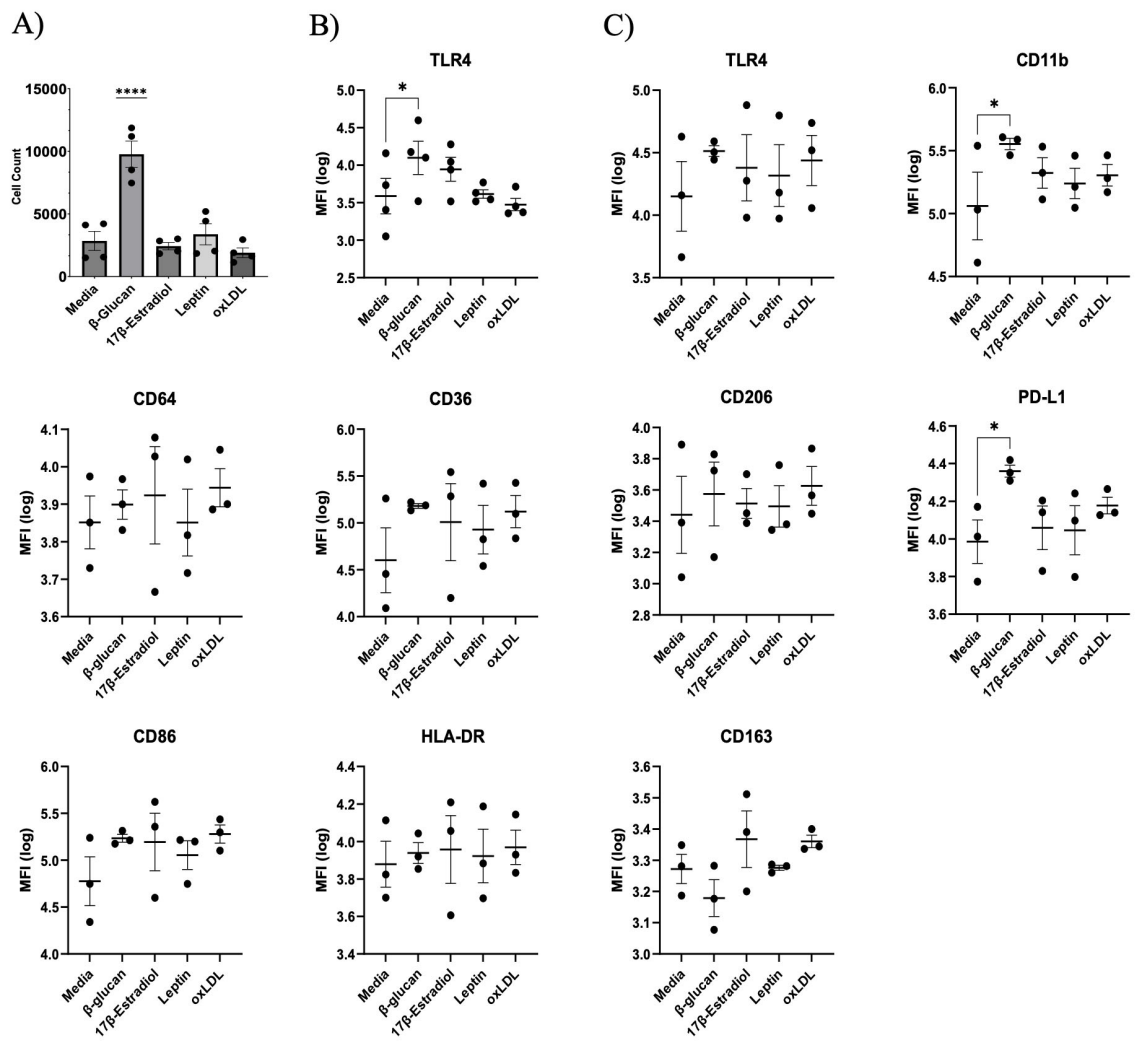
β-glucan increases human monocyte cell counts and expression of specific surface markers. Monocytes were incubated for 24 hours with media alone, or media with 5 µg/mL β-glucan, 330 pg/mL 17β-estradiol, 100 ng/mL leptin or 10µg/mL oxLDL and then rested for five days with fresh media on day three. On day six, monocytes were either **(A)** DAPI stained and nuclei counted using the PICO instrument, or **(B)** rechallenged with 25 ng/mL LPS for 24 hours and then stained for TLR4, or **(C)** rechallenged with SK-MEL-28 (9.02 x 10^3^ cells per well) in transwells for 24 hours and then stained for TLR4, CD11b, CD206, PD-L1 CD86, HLA-DR, CD163, CD36 and CD64. **(B, C)** Fluorescence was measured using an Aurora flow cytometer. Geometric mean fluorescence intensity (MFI) for each fluorophore was calculated and log10 transformed. Representation of monocyte purity indicated in **Supplementary Material 1**. Data obtained using three **(C)** or four **(A, B)** separate donors are shown as scatter plots with mean (bar) and SEM indicated. Statistical analysis was performed using RM one-way ANOVA and Dunnett’s multi-comparison test to compare treatments with media alone; *P < 0.05, ****P<0.0001.

Previous *in vitro* studies have reported that exogenous priming of monocytes can increase both cell numbers and Toll-like Receptor 4 (TLR4) expression in response to secondary LPS challenge (7, 27, 44). Consistent with those results, our *in vitro* study showed β-glucan priming increased human monocyte numbers (**Figure 2A**), and TLR4 expression in response to secondary LPS challenge (**Figure 2B**). We then analyzed the expression of surface markers in primed monocytes from three donors following secondary challenge with SK-MEL-28 melanoma cells rather than LPS. Priming with β-glucan significantly increased CD11b and PD-L1 expression compared to cells not primed (**Figure 2C**). The remaining surface markers did not show any significant differences in presentation. Monocytes primed with endogenous stimuli (17β-estradiol, leptin and oxLDL) exhibited no significant changes in surface marker presentation compared to untreated cells.

### OxLDL priming enhances superoxide production and MMP9 activity of monocytes

The functional capacity of primed monocytes was assessed by measuring superoxide generation and matrix metalloprotease 9 (MMP9) activity. Superoxide is produced in monocytes by NADPH oxidase, a membrane-bound multicomponent enzyme that assembles at the time of activation (45). The extracellular release of superoxide was detected by the reduction of cytochrome C, which is a method specific for superoxide because the inclusion of catalase eliminates activation of cellular peroxidases (36, 46).

**Figure 3:**
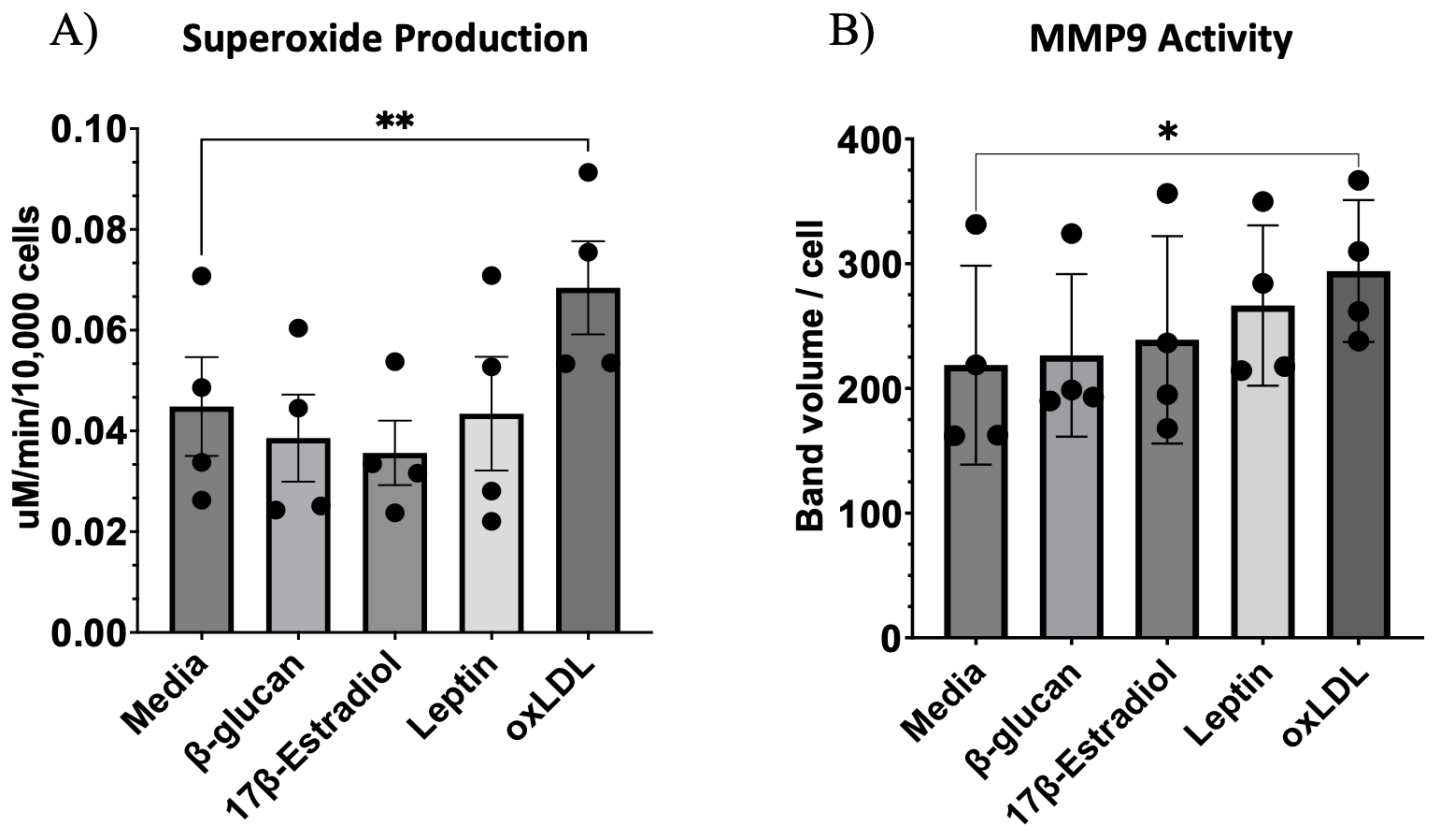
OxLDL primed monocytes exhibit enhanced functional activity. Monocytes were incubated for 24 hours with media alone, or media with 5 µg/mL β-glucan, 330 pg/mL 17β-estradiol, 100 ng/mL leptin or 10µg/mL oxLDL and then rested for five days with fresh media on day three. On day six, monocytes were: **(A)** restimulated (1h) with 50 ng/mL PMA, in the presence of cytochrome C and catalase, and monitored for superoxide production, which was normalized by cell count. **(B)** rechallenged with 25 ng/mL LPS for 24 hours in VP-SFM media. Conditioned media was collected and analyzed using zymography. Zymograms were imaged using an Q9 Alliance Imager, and quantification of degradation bands was performed using Nine Alliance Q9 software. The volume of degradation bands was normalized by cell count. **(A, B)** Data was obtained using four separate donors for each graph, which are shown as a scatter plot with mean (bars) and SEM indicated. Statistical analysis was performed using either **(A)** RM one-way ANOVA and Dunnett’s multi-comparison test or **(B)** Friedman test and Dunn’s multi-comparison test to compare treatments with media alone; *P < 0.05, **P<0.01.

Monocytes primed with oxLDL demonstrated significant increase of superoxide production when stimulated by phorbol 12-myristate 13-acetate (PMA). There was a rate increase in the PMA response from 0.045 µM/min/10,000 cells in untreated cells to 0.068 µM/min/10,000 in oxLDL primed monocytes (**Figure 3A**). No significant changes were observed in monocytes primed with β-glucan, 17β-estradiol or leptin.

It has been reported that monocyte priming upregulates expression of MMP9 in response to secondary challenge, but whether this translates to increased matrix degradation remains unknown (19). Using zymography, the presence and conversion of pro-MMP9 into enzymatically active MMP9 was assessed. oxLDL primed monocytes demonstrated a significant increase in gelatin degradation, indicative of increased MMP9 secretion and/or activation, in response to subsequent stimulation by LPS (**Figure 3B**). The other priming stimulants used did not induce significant changes in relative MMP9 activity.

### Primed monocytes retain transcriptomic changes

Reports of gene expression profile modifications within primed monocytes are becoming increasingly frequent as studies investigate the priming capacity of different stimulants (7, 19, 47). Innate priming is clearly associated with changes in gene expression. However, both the extent of these changes and the degree to which they are retained within resting monocytes after primary stimulation remains underexamined. Here, monocytes were incubated with media, or media with 17β-estradiol, leptin or oxLDL for 24 hours and then the cells were rested for five days, before RNA was extracted for sequencing on the sixth day.

**Figure 4:**
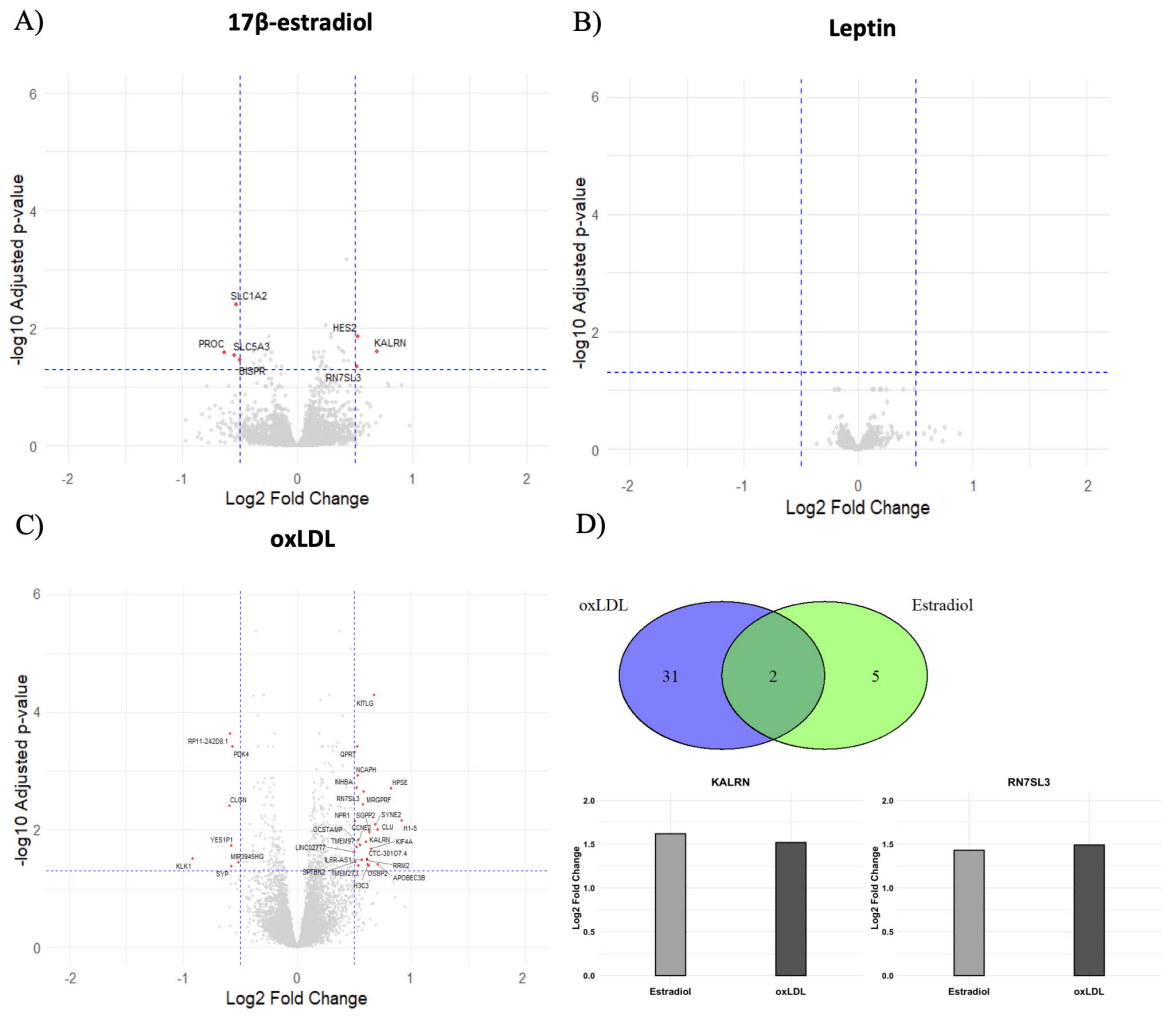
Differentially expressed genes between primed monocytes and untreated control monocytes. Monocytes were incubated for 24 hours with media alone, or media with 330 pg/mL 17β-estradiol, 100 ng/mL leptin or 10µg/mL oxLDL and then rested for five days with fresh media on day three. On day six, RNA was extracted and sequenced. Comparisons were made between media and priming condition (17β-estradiol, leptin or oxLDL) pooled (across donors) differential gene expression, which are plotted as a volcano plot. A nominal threshold of log2 fold change >0.5 or < -0.5 (vertical blue lines) and adjusted p ≤ 0.05 (horizontal blue line) was used to define differentially expressed genes. Data for each graph was obtained from 10 separate donors and the Wald test using Benjamin-Hochberg correction tested significance of differential expression. **(A–C)** Grey dots show gene expression values that did not reach significance and/or threshold of fold change. Gene names are listed for red dots that reached significance. **(D)** Overlap of differential gene expression within 17β-estradiol and oxLDL primed monocytes and corresponding fold change in expression.

Analysis of gene expression in monocytes primed with 17β-estradiol and monocytes cultured in media alone identified seven significant differentially expressed genes (**Figure 4A**). Three genes were upregulated (*HES2, KALRN, RN7SL3)* and four genes (*SLC1A2, PROC, SLC5A3, BISPR)* were downregulated (**Supplementary Material 2**). Examination of gene expression in monocytes primed with leptin showed no significant differentially expressed genes when compared with monocytes cultured in media alone (**Figure 4B**). Comparatively, analysis of gene expression in monocytes primed with oxLDL and monocytes cultured in media alone identified 33 genes with significant differential expression (**Figure 4C**). Of these genes, 26 genes were upregulated, while the remaining 7 were downregulated (**Supplementary Material 2**).

Two genes (*KALRN* and *RN7SL3*) were differentially expressed when monocytes were primed with either 17β-estradiol or oxLDL (**Figure 4D**). Both genes were found to be upregulated to similar extents by the priming treatments, with *KALRN* and *RN7SL3* exhibiting a fold change of 1.62 and 1.43, respectively, in monocytes primed with 17β-estradiol, and fold changes of 1.52 and 1.49, respectively, in monocytes primed with oxLDL.

### Priming monocytes changes global DNA methylation

DNA methylation is one of the key epigenetic mechanisms that regulate gene expression, occluding promoter sites from transcriptional machinery when present (48). However, its role in endogenous innate priming of human monocytes is largely unknown.

Using mass spectrometry, measurement of 5-methylcytidine (5mC), 5-hydroxymethylcytidine (5hmC) and deoxycytidine is indicative of global DNA methylation levels. The presence of 5mC demonstrates DNA methylation, 5hmC indicates that the process of demethylation of DNA is occurring, while deoxycytidine is representative of no methylation on the DNA (49).

**Figure 5:**
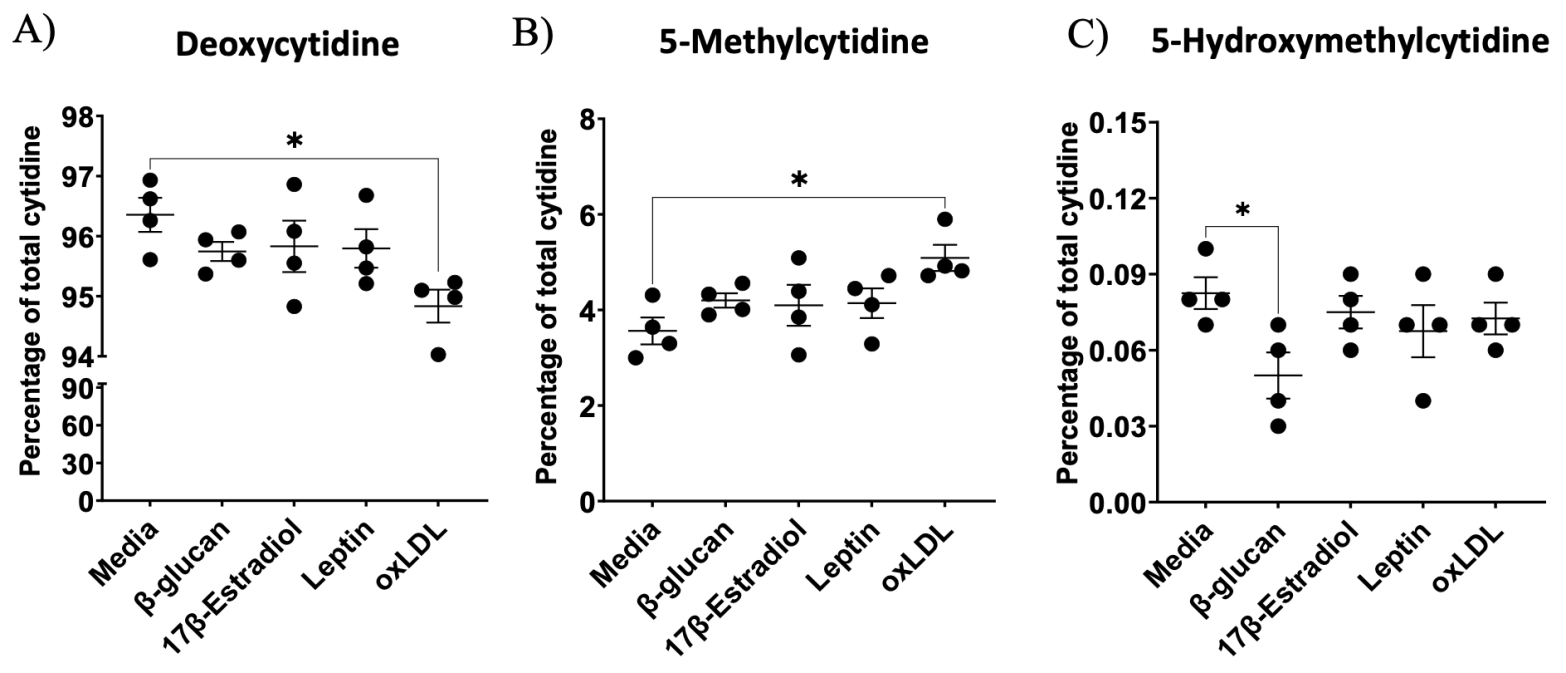
Primed monocytes exhibit altered global deoxycytidine, 5-methylcytidine and 5-hydroxymethylcytidine levels. Monocytes were incubated for 24 hours with media alone, or media with 5 µg/mL β-glucan, 330 pg/mL 17β-estradiol, 100 ng/mL leptin or 10µg/mL oxLDL and then rested for five days with fresh media on day three. On day six, DNA was extracted from monocytes and nucleotides were digested. Mass spectrometry was used to measure **(A)** deoxycytidine, **(B)** 5-methylcytidine and **(C)** 5-hydroxymethylcytidine, and global concentrations were calculated from standard curves and converted to percentages of total cytidine. Data was obtained using four separate donors for each graph, which are shown as a scatter plot with mean (bar) and SEM indicated. Statistical analysis was performed using RM one-way ANOVA and Dunnett’s multiple comparison test to compare treatments with media alone; *P < 0.05.

Monocytes primed with oxLDL significantly downregulated deoxycytidine levels (**Figure 5A**) and upregulated 5mC levels (**Figure 5B**). In addition, monocytes primed with β-glucan showed significantly downregulated global levels of 5hmC, compared to monocytes cultured with media alone (**Figure 5C**). In contrast, monocytes primed with 17β-estradiol or leptin did not exhibit significant changes in deoxycytidine, 5mC or 5hmC levels.

## Discussion

Recent research has shown that primary stimulants (e.g. β-glucan) can prime innate immune cells through metabolic and epigenetic regulation, leading to long-lasting changes in phenotype and effector function (50). This in turn can result in a heightened or dampened response to a secondary stimulus (6–9). Previous studies have shown that endogenous compounds including oxLDL can prime monocytes, and that WGP can improve monocyte inflammatory responses in the context of tumor immune response (7, 9). However, the phenotypical and functional characteristics of endogenously primed monocytes, as well as the transcriptomic and epigenetic changes driving these characteristics remain underexamined.

The impact of monocyte priming on the surface marker phenotype observed following secondary challenge by indirect contact with melanoma cells was assessed. Monocytes primed with β-glucan were found to increase expression of CD11b and PD-L1 following secondary stimulation with melanoma cells. The upregulation of CD11b observed in the current study is consistent with previous reports of increased CD11b presentation in β-glucan primed monocytes following secondary LPS stimulation (51). CD11b modulates multiple aspects of monocyte functional responses such as migration, phagocytosis, as well as the FcγR and TLR signaling pathway (52–55). Within tumors, CD11b upregulation on monocytes has been suggested to promote anti-tumor responses, suggesting monocyte priming with β-glucan may promote a more favorable anti-tumor phenotype (56). However, these findings must be interpretated with caution given the small number of experimental repeats (n=3) performed. In this study, a melanoma cell line was utilized as a secondary stimulant as melanoma is a relatively immunogenic cancer and widely used as an immunotherapy model (57). Whether the same effects of innate priming occur in the context of other less immunogenic cancers merits further investigation.

The regulatory marker PD-L1 is a potent inhibitor of T cell activity, and its upregulation has been associated with a more immuno-suppressive monocyte phenotype (58, 59). However, recent literature has demonstrated that PD-L1^+^ TAMs can be immunostimulatory and mature compared to PD-L1^-^ cells (60). It is clear that the functional impact of PD-L1 is context dependent, warranting further investigation, particularly as it is a direct and indirect target of immunotherapy (58–65).

Previous studies have widely characterized cytokine production in primed monocytes, but alteration in function remains to be fully investigated (7, 19, 66, 67). Here, the rate of production of superoxide and MMP9 activity were assessed in primed monocytes exposed to secondary challenge. Traditionally, ROS generation is used to assess monocyte function. However, such measurements reflect overall levels rather than those of each individual species, which in turn limits insight into their biological effect and underlying mechanisms (7, 68, 69).

Superoxide is produced by immune cells and can form other reactive species, which regulate immune response to disease (66). Here, oxLDL priming was found to increase the rate of superoxide production by monocytes in response to secondary PMA stimulation. PMA induces superoxide production through stimulation of NADPH oxidase assembly (45). Therefore, upregulation of superoxide may indicate increased transcription of NADPH oxidase enzymes and/or increased substrate/co-factors for enzyme activity (70, 71).

Monocytes primed with oxLDL showed an increase in collagen degradation following secondary challenge with LPS. This is consistent with increased production and/or activity of MMP9. The protein MMP9 is a proteolytic metalloenzyme belonging to the gelatinase family that degrades extracellular matrix, particularly type IV collagen, allowing for monocyte movement through tissue (72–74). It has been previously reported that oxLDL priming of monocytes increases MMP9 expression while, in contrast, β-glucan priming downregulates MMP9 expression (19). Our observation that β-glucan did not modulate MMP9 may reflect the fact we measured MMP9 activity rather than expression levels. Increased MMP9 activity in oxLDL primed monocytes may also align with our finding that oxLDL increases superoxide production, as mitochondrial ROS induced by NOX4 have previously been associated with increased stability of MMP9 mRNA (75). This may suggest an alternative role for superoxide in modulating function within primed monocytes, further demonstrating the need to investigate reactive oxygen species separately.

With the identification of altered phenotype and function in primed monocytes, we examined changes in transcription and DNA methylation profiles in resting monocytes that were primed for 24 hours and then allowed to rest in fresh media for five days. In monocytes that were primed with 17β-estradiol, transcriptomic analysis identified seven genes that were differentially expressed. Grouping of annotations for these genes suggests modulation of pathways including protein modification and movement, immune function and amino acid metabolism (76–81). In particular, upregulation of *KALRN* has been associated with proliferation, angiogenesis, secretory granule release, migration and energy metabolism, which would support the metabolic needs of primed cells (10, 11, 82). Additionally, *7SL* RNA genes, such as *RN7SL3*, encode for the major RNA component of the signal recognition particle (SRP) complex, a ribonucleoprotein particle responsible for transporting nascent secretory and membrane proteins to the endoplasmic reticulum (77–80). Considering the role of SRP in targeting and transporting approximately a third of total cellular proteins, its upregulation is likely a necessary upstream component of numerous cellular pathways in primed monocytes (78).

Priming monocytes with oxLDL induced significant differential expression of 33 genes. Grouping annotations of genes indicated regulation of pathways including metabolism, immune response, protein transport and modification (77–80, 82, 83). Of note, upregulation of *HPSE* may indicate increased migration potential through the extracellular matrix as it is required for the cleavage of heparan sulphate proteoglycans (83). Upregulation of monocyte migration capacity agrees with previous studies that showed oxLDL priming of monocytes increases expression of proteins involved in matrix degradation (MMP2 and MMP9) after secondary challenge (19). Heparanase has also been reported to increase expression of MMP9, providing a potential mechanism for the upregulated MMP9 expression previously reported after monocyte priming (19, 84).

Monocytes primed with β-glucan were identified with decreased levels of 5hmC, while monocytes primed with oxLDL had decreased deoxycytidine levels and increased presence of 5mC. Decreased levels of 5hmC suggest either increased passive demethylation as cells proliferate and/or increased activity of enzymes including demethylation protein ten-eleven translocation protein 2 (TET2), which convert 5hmC into 5-formylcytosine (5fC) and then 5-carboxylcyosine (5caC) (85–88). Increased levels of 5mC, and correspondingly, decreased levels of deoxycytidine within oxLDL primed monocytes are indicative of a more differentiated and silent genome (49). This increase in levels of 5mC suggests increased activity of DNA methyltransferases, which mediate cytosine residue acceptance of methyl groups donated by *S*-adenosyl methionine (49). Our results provide direct evidence that endogenous innate priming influences global DNA methylation. Future studies should determine which Cytosine-phosphate-Guanine (CpG) islands possess altered methylation, and in turn, which genes are being regulated as a result of priming events (49). Additionally, this opens the possibility of regulating innate priming through supplementation with compounds such as vitamin C, which has previously been demonstrated to upregulate activity of the demethylation enzyme TET2 (89).

Interestingly, monocytes primed with leptin did not demonstrate any observable changes in the current study. This was unexpected, as a previous study demonstrated that leptin priming can augment cytokine production in response to secondary challenge (90). However, that study used a leptin concentration of 50 ng/mL, while the current study exposed cells to 100 ng/mL, suggesting a potential difference in dose response (90). Here, we selected a concentration of 100 ng/mL, as this concentration was shown to stimulate monocytes *in-vitro* and was physiologically relevant in obesity (23, 30, 31). Differences in dose have previously been reported with primary stimulation by LPS, which has been demonstrated to induce innate immune cell training at low concentrations and immune tolerance at high concentrations (91). Further investigation into the capacity of leptin to induce priming at different concentrations is warranted.

Our study had several limitations including the use of fetal bovine serum (FBS) in place of human serum (92). Additionally, the use of bulk RNA sequencing masked heterogeneity within monocyte populations, limiting examination of expression profiles within phenotypes of interest (93). Similarly, analysis of global DNA methylation levels also limited insight into which genes possessed the alterations in methylation, and future studies require more targeted techniques (e.g. CHIP-methylation arrays) to obtain site specific modifications (94). In the current study, a number of experiments, including those related to melanoma driven phenotypic changes, were performed a limited number of times. Therefore, these results, although interesting, must be interpreted with caution and require further investigation. In addition, only three biological repeats were performed in a portion of experiments, limiting strength of conclusions. This study was designed to investigate the acute effects of endogenous obesity-associated factors on monocyte priming. For this reason, monocytes were isolated from healthy, non-obese males. However, this limited translatability as cells were exposed to singular stimulants. Comparison with monocytes isolated from obese individuals would be informative when assessing phenotype and function in monocytes exposed to obesity-associated compounds. Women were not included in this study, as one of the investigated compounds was 17β-estradiol. Therefore, further studies are required to confirm the external validity of our findings in clinically relevant populations.

Results from this study confirm that endogenous compounds associated with obesity can prime monocytes to exhibit altered phenotype and function. Transcriptomic analysis revealed that monocytes primed with 17β-estradiol and oxLDL exhibited differential gene expression compared to untreated monocytes, with implications for pathways related to metabolism, protein regulation and immune response. A key finding from this study was that monocytes primed with oxLDL exhibit altered global DNA methylation, providing direct evidence to support DNA methylation as a mechanism of endogenous priming in human monocytes. Together, results from this study demonstrate that obesity-related endogenous compounds 17β-estradiol and oxLDL are capable of priming human monocytes, resulting in a modified phenotype and functional response to secondary challenge.

## Data Availability

The original contributions presented int he study are included in the article/**Supplementary Material**, further inquiries can be directed to the corresponding author/s.
