# Supplementary material for: Priming of human monocytes by β-glucan and obesity-associated factors: modifications to global DNA methylation, gene expression, phenotype and function
Source: Front Immunol. 2026 Jul 20;17:1825431. doi: 10.3389/fimmu.2026.1825431 (PMC13429425; doi:10.3389/fimmu.2026.1825431)
Supplement: Supplementary file 1 [file DataSheet1.docx]

**Supplementary Material 1**

**D)**

**A)**

**C)**

**B)**


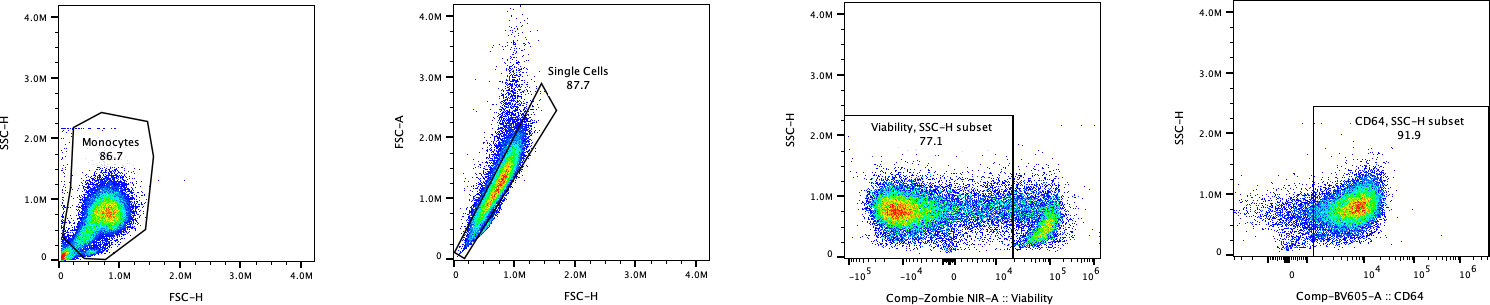


**Figure 1:** Gating strategy for phenotypic analysis of monocyte populations. Following (A) gating of monocyte populations based on forward versus side scatter plots, (B) forward side scatter area versus forward side scatter height were used to select single cells. Plots of (C) forward side scatter versus Zombie NIR were then used to gate on viable cells which were in turn analysed with respect to expression of fluorescent markers including CD64 (D). Unmixed spectral data was analysed using FlowJo software.
